# Ocular Complications of Myopia: Bibliometric Analysis and Citation Networks

**DOI:** 10.3390/reports6020026

**Published:** 2023-06-01

**Authors:** Miguel Ángel Sánchez-Tena, Clara Martinez-Perez, Cesar Villa-Collar, Cristina Alvarez-Peregrina

**Affiliations:** 1 Department of Optometry and Vision, Faculty of Optics and Optometry, Universidad Complutense de Madrid, 28037 Madrid, Spain; cristina_alvarez@ucm.es; 2 ISEC LISBOA—Instituto Superior de Educação e Ciências, 1750-179 Lisbon, Portugal; clara.perez@iseclisboa.pt; 3 Faculty of Biomedical and Health Science, Universidad Europea de Madrid, 28670 Madrid, Spain

**Keywords:** myopia, citation network, ocular complications, glaucoma, retinal detachment, maculopathy

## Abstract

Background: The objective of this study is to determine the relationship between publications and authors. In turn, the different areas of research on the ocular complications of myopia are also identified, and the most cited publication by citation networks is determined. Methods: The search for publications is carried out in the Web of Science database using the term “myopia OR nearsightedness AND retin* OR degenerat* OR detachment OR patholog* OR glaucoma OR cataract OR machulopathy OR “choroidal neovascularization” for the period between 1978 and December 2022. Publication analysis was performed using the Citation Network Explorer, VOSviewer and CiteSpace software. Results: A total of 9357 publications and 78,400 citation networks were found across the network, with 2021 being the year with the highest number of publications, 712. The most cited publication was “Myopia”, published in *The Lancet* journal in 2012 by Morgan et al. with a citation index of 1006. By using the clustering function, seven groups covering the different research areas in this field were found: axial growth of the eye; myopic maculopathy; ocular complications in patients with myopia after a surgery; glaucoma; retinal detachment; cataracts and retinopathy of prematurity. Conclusions: The citation network offers a quantitative and qualitative analysis of the main papers on ocular complications of myopia. The research on this field is multidisciplinary; however, the main topic studied is the axial growth of the eye.

## 1. Introduction

Myopia is considered to be a public health problem worldwide, with a prevalence of more than two billion people (28.3% of the global population), of whom 277 million people (4.0%) have high myopia [1]. The International Institute of Myopia has defined pathologic myopia as an excessive growth of the axial length associated with myopia, which leads to structural changes in the posterior segment of the eye and can also lead to a loss of best-corrected visual acuity [2]. It should be noted that the term “pathologic myopia” tends to be confused with “high myopia”. However, they are distinctly different. High myopia is a high degree of myopic refractive error, whereas pathologic myopia is defined by a presence of typical complications in the fundus (back of the eye) [3]. Duke Elder defined pathologic myopia as “myopia with degenerative changes especially in the posterior segment” [4].

Pathological myopia is present in 3% of the world population, although there are differences in its prevalence rate [5]. Between 1% and 3% of Asians and 1% of Caucasians have pathological myopia [3]. It tends to cause problems of vision or blindness in 0.2% to 1.5% of Asians and 0.1% to 0.5% of Caucasians [5]. For this reason, it is considered one of the main causes of low vision in the working age and elderly populations. Thus, in Taiwan, the second leading cause of visual impairment in people over 65 years of age is pathological myopia [6]. In Japan, it is the third leading cause of bilateral low vision and the leading cause of monocular blindness in people over 40 years of age [7], and in China, it is the leading cause of blindness and low vision in people aged 40 to 49 years [8]. As for the Western countries, it is the third leading cause of blindness according to the Rotterdam Study, the Copenhagen City Eye Study and the Los Angeles Latino Eye Study [9,10,11].

The main complication and cause of visual impairment from pathological myopia is myopic macular degeneration. The main features are lacquer cracks, Fuchs spot, choroidal neovascularisation and chorioretinal atrophy [12]. Posterior staphyloma is also sometimes considered a specific type of myopic macular degeneration, although some consider it more of a risk factor for developing it [12]. The prevalence ranged from 0.2% in rural central India to 1.2% in Caucasian Australians to 4.0% in the Singapore Eye Disease Epidemiology study. When classifying its prevalence according to the degree of myopia, the prevalence ranged between 13.3% and 65.4% in high myopia, between 0.3% and 7.8% in moderate myopia, and between 0.1% and 7.0% in low myopia [13,14,15,16,17,18,19]. The best corrected visual acuity was found to be more affected in eyes with myopic macular degeneration than in eyes without that pathology [17,18,20]. Macular atrophy had the greatest impact on visual acuity, followed by choroidal neovascularisation, irregular atrophy, diffuse atrophy or lacquer cracks based on a longitudinal study of patients with myopic macular degeneration in Japan. Patients with only a tiger-striped eye background did not have a decrease in visual acuity [21]. Other studies have also shown that patients with macular atrophy, choroidal neovascularisation or a Fuchs spot had poorer visual acuity compared to those with irregular or diffuse atrophy, lacquer cracks or tiger-striped eye background. Progression to more severe stages of MMD was more frequent in older patients [20,21,22,23].

Regarding cataracts, a nuclear cataract can result in a myopic shift, making it difficult to determine the original refractive error [12]. It has been found that the risk of retinal detachment in myopic people ranged between 0% and 3.84% [24]. Various studies have shown an increased risk of retinal detachment in myopic patients after surgery. This increase was greater in patients under 55 years of age [25,26,27].

Regarding open-angle glaucoma (OAG), in 1982, Perkins et al. [27] reported a higher percentage of myopic patients in the population with this type of glaucoma. In a meta-analysis based on a group of 11 patients, the authors also showed that myopic people have a higher risk of OAG [28]. For the diagnosis of open-angle glaucoma, most studies have been based on visual field defects and optic disc aberrations, thus demonstrating the strong association that exists for any myopia compared to emmetropia. As the degree of myopia increased, this association became stronger.

The objective of this study was to identify the research areas and the publication with the highest number of citations about complications associated with myopia. In addition, the associations that exist between the publications and the different research groups were analysed.

Through the analysis of citation networks, other relevant publications were obtained to show qualitatively and quantitatively the connections between articles and authors by creating groups [29]. This also allowed quantifying the publication with the highest number of citations within each group, as well as study the development of a field of research or focus the bibliographic search on a specific topic [30,31].

## 2. Materials and Methods

### 2.1. Database

The search for publications was carried out in the Web of Science (WoS) database using the following search terms: (myopia OR nearsightedness) AND (retin* OR degenerat* OR detachment OR patholog* OR glaucoma OR cataract OR machulopathy OR “choroidal neovascularization”).

The following citation indexes were used: Social Sciences Citation Index, Science Citation Index Expanded and Emerging Sources Citation Index. The selected time interval for the search was from 1978 to December 2022. The publications were searched and downloaded on 23 January 2023.

### 2.2. Data Analysis

The Citation Network Explorer software was used to analyse the publications and visualise the citation networks. This approach has allowed, starting from a citation network with several million publications, performance of a deeper analysis; as a result, smaller citation networks could be obtained.

Also using the citation score attribute, a quantitative analysis of the publications with the most citations was performed. Thus, apart from the internal connections within the Web of Science database, other external connections, that is, other databases, were also analysed [31].

Subsequently, the most connected publications were assigned to the same group using the clustering function. The clustering functionality was achieved by using the formula developed by Van Eck in 2012 [31].

$$V\left(c_{1\;},\ldots ,c_{n}\right)=\underset{i<j}{\sum }\delta \;\left(c_{i},c_{j}\right)\left(s_{ij}-\gamma \right).$$

The publications considered to be the core of a citation network were analysed using the Identifying Core Publications functionality. This study considered publications with four or more citations in the citation network. The higher the value of this parameter, the lower the number of core publications [31]. On the other hand, it was possible to analyse each of the groups at different levels through the drill-down function.

Besides this, the CiteSpace v. 5.6.R2 software (Chaomei Chen, College of Computing and Informatics, Drexel University, PA, USA) was used to perform scientometric analyses by establishing certain parameter indicators, with the H index used to evaluate the quantity and level of academic output of researchers or academic institutions [32]. The degree indicated the number of connections among the authors (organisations, countries) in the co-occurrence knowledge graph; consequently, a higher value in this degree indicated that there was a greater level of communication and collaboration. Furthermore, the centrality value measured the importance of the nodes within the research cooperation network, while the half-life was a parameter representing the continuity of institutional research from a time perspective [33].

## 3. Results

The first articles on the various complications associated with myopia were published in 1978. However, since 2000, the number of publications has increased exponentially (1978–1999: 10.6%; 2001–2022: 89.4%). After the WoS search, 9357 publications were found in all fields, as well as 78,400 citation networks.

**Figure 1:**
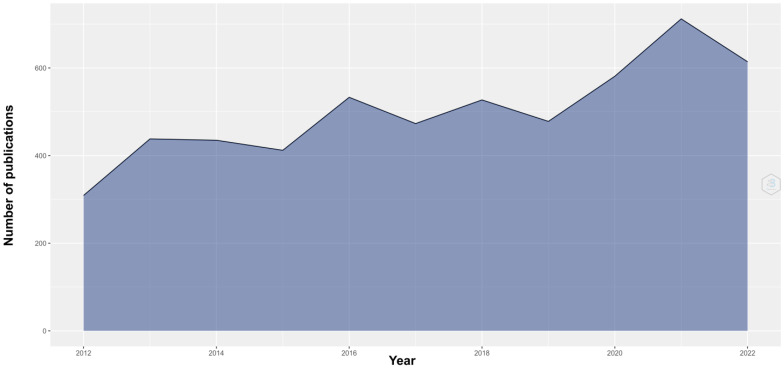
Number of publications in the last 10 years; 2021 was the year with the most citations.

The year with the highest number of publications was 2021, with 712 publications and 137 citation networks (Figure 1).

### 3.1. Description of the Publications

Of all the publications, 84.5% were articles, 6.3% were reviews, 5.2% were records and 4.0% were abstracts.

#### 3.1.1. Languages and Countries

**Figure 2:**
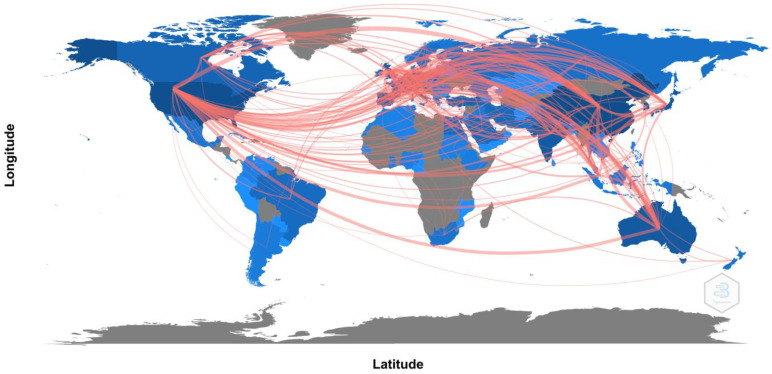
World map on connections between countries. China and USA are the countries with the most connections.

With regard to the language of the publications, 95.6% were in English, 1.8% were in German and 1.5% were in French. Geographically, as shown in Figure 2, the countries with the highest number of publications and connections with other countries were the United States (publications: 2217; degree: 135; half-life: 32.5; connections: 47,789), China (publications: 1610; degree: 63; half-life: 24.5; connections: 39,774) and Japan (publications: 798; degree: 50; half-life: 35.5; connections: 24,744).

This is a multidisciplinary research area, and the fields of ophthalmology (78.4%) and surgery (8.0%) are particularly noteworthy.

#### 3.1.2. Authors and Institutions

**Figure 3:**
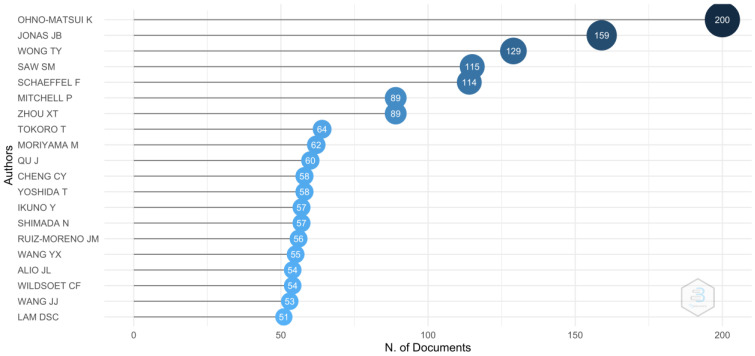
Top 20 authors with the highest number of publications. Ohno-Matsui K is the author with the most citations.

The authors with the highest number of publications (Figure 3) were K. Ohno-Matsui (publications: 2.1%; degree: 188; connections: 24,108), J.B. Jonas (publications: 1.7%; degree: 147; connections: 16,444) and T.Y. Wong (publications: 1.4%; degree: 138; connections: 8305).

The institutions with the highest number of publications (Table 1) were the University of California System (3.4%), National University of Singapore (3.0%) and Singapore National Eye Center (3.0%).

#### 3.1.3. Journals

Table 2 shows the main journals and the number of publications in each journal according to the WoS database.

#### 3.1.4. Keywords

Additionally, the most commonly used keywords were “myopia”, “prevalence” and “eyes”. Table 3 shows the most-used keywords from the most relevant publications.

### 3.2. Most Cited Publications

**Figure 4:**
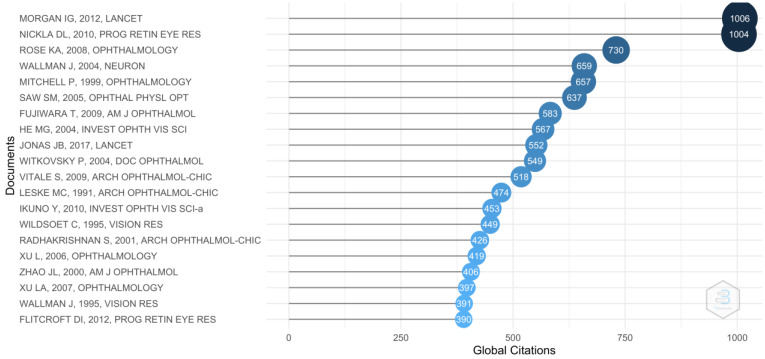
Top 20 most cited publications. The most cited publication is from Morgan et al. in 2012.

As shown in Figure 4, the most cited article was “Myopia” published in *The Lancet* journal in 2012 by Morgan et al. [34]. This publication reviews the prevalence of myopia worldwide, highlighting the high rates of this condition in East Asia. At the same time, it analyses the risk factors and pathologies associated with high myopia.

### 3.3. Clustering

The clustering function identified ten groups, seven of which had a significant number of publications in order to establish relationships amongst publications. The colour of the article represents the group they belong to and the lines between elements represent bonds. In other words, each group has a different colour, and the lines are the unions with other groups. The size of the circles depends on the number of citations. The larger the circle, the larger the citations.

**Figure 5:**
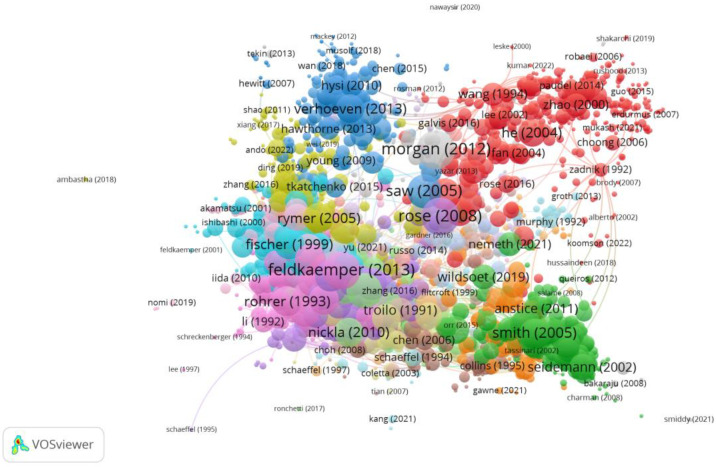
Citation network in Group 1. The largest circle and therefore the most cited publication was the article by Morgan et al. in 2012 [34]. Within this group, there are 19 subgroups, each with a different colour.

In Group 1, there were 2227 publications and 25,464 citations found across the whole network. The most cited publication was the article by Morgan et al. [34] which was published in 2012 in *The Lancet.* This was also the most cited publication out of the 20 most cited publications. The articles in this group analysed the axial growth of the eye and its relationship with the degree of myopia (Figure 5).

**Figure 6:**
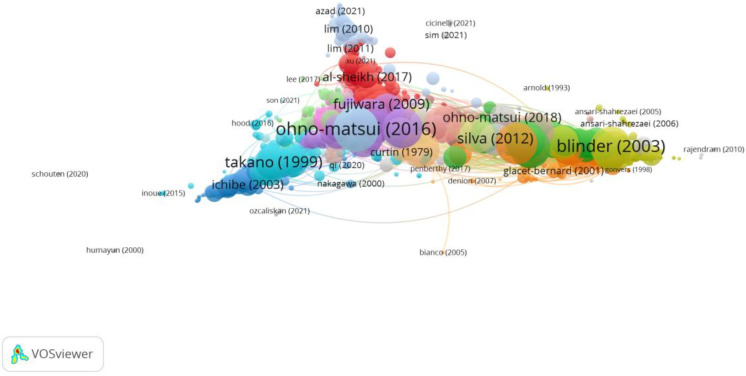
Citation network in Group 2. The largest circle and therefore the most cited publication was the article by Ohno-Matsui et al. in 2016 [35]. Within this group, there are 22 subgroups, each with a different colour.

Group 2 was made up of 1872 publications and 19,237 citation networks. The publication by Ohno-Matsui et al. [35] in 2016 in the *American Journal of Ophthalmology* was the one with the highest number of citations. The articles in this group analyse the impact on vision and quality of life, as well as the treatment methods for myopic maculopathy and choroidal neovascularisation (Figure 6).

**Figure 7:**
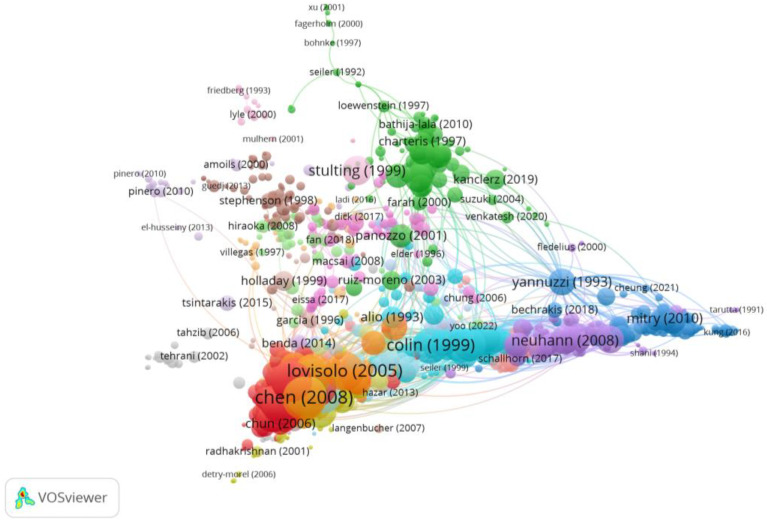
Citation network in Group 3. The largest circle and therefore the most cited publication was the article by Chen et al. in 2008 [36]. Within this group, there are 20 subgroups, each with a different colour.

Group 3 was made up of 1134 publications and 7127 citation networks. The most cited publication was the one by Chen et al. [36] in the *Journal of Cataract and Refractive Surgery* in 2008. The articles in this group analyse the risk of suffering ocular complications in patients with myopia after undergoing refractive surgery or anterior chamber intraocular lenses (Figure 7).

**Figure 8:**
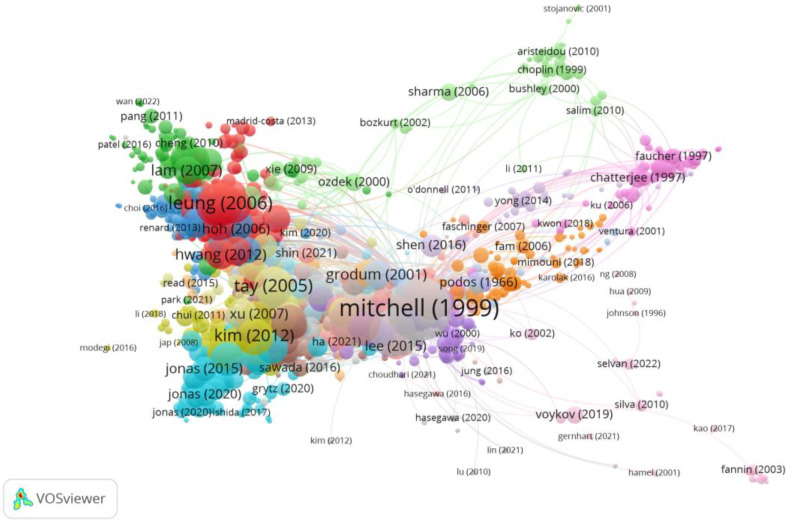
Citation network in Group 4. The largest circle and therefore the most cited publication was the article by Mitchell et al. in 1999 [37]. Within this group, there are 25 subgroups, each with a different colour.

Group 4 was made up of 1122 publications and 7848 citation networks. The most cited publication was the one by Mitchell et al. [37] in *Ophtalmology* in 1999. The articles in this group analyse the risk of developing glaucoma in myopic patients, and treatment methods (Figure 8).

**Figure 9:**
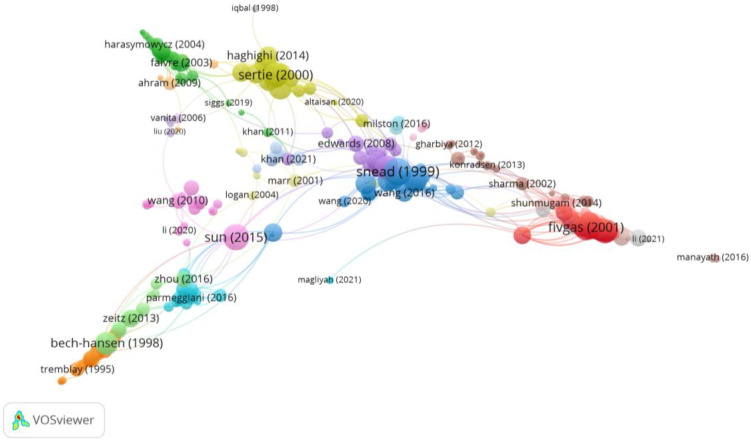
Citation network in Group 5. The largest circle and therefore the most cited publication was the article by Snead et al. in 1999 [38]. Within this group, there are 21 subgroups, each with a different colour.

Group 5 was made up of 385 publications and 1271 citation networks. The most cited publication was the one by Snead et al. [38] in the *Journal of medical genetics* in 1999. The articles in this group analyse the syndromes and genetic changes associated with myopia, as well as the risk and treatment methods for retinal detachment and damage (Figure 9).

**Figure 10:**
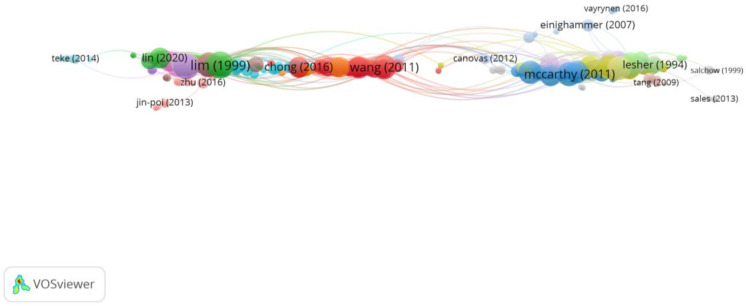
Citation network in Group 6. The largest circle and therefore the most cited publication was the article by Lim et al. in 1999 [39]. Within this group, there are 15 subgroups, each with a different colour.

Group 6 was made up of 373 publications and 1662 citation networks. The most cited publication was the one by Lim et al. [39] in *Investigative Ophthalmology & Visual Science* in 1999. The articles in this group analyse the association between myopia and the development of cataracts (Figure 10).

**Figure 11:**
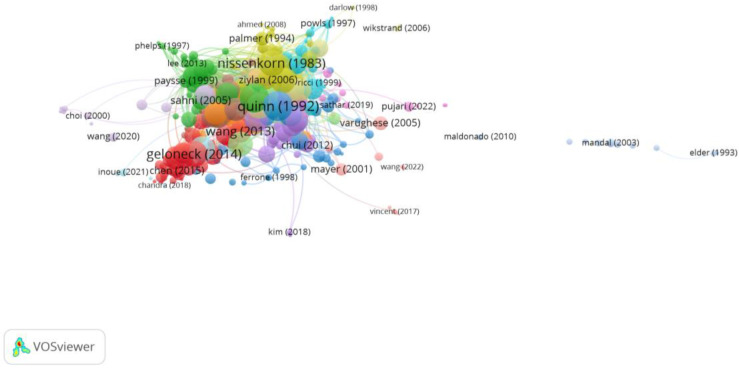
Citation network in Group 7. The largest circle and therefore the most cited publication was the article by Quinn et al. in 1992 [40]. Within this group, there are 15 subgroups, each with a different colour.

Group 7 was made up of 328 publications and 2523 citation networks. The most cited publication was the one by Quinn et al. [40] in *Ophthalmology* in 1992. The articles in this group analyse the incidence and risk factors associated with retinopathy of prematurity (Figure 11).

## 4. Discussion

In this study, a bibliometric and citation network analysis has been carried out on the ocular complications associated with myopia. In order to select the keywords, we have used other articles, bibliometrics and meta-analyses as our basis [12,40,41,42,43]. The number of publications in this area has increased since 2000, mainly in the last 10 years. This coincides with the study by Shan et al. [41], in which the authors used a bibliometric analysis to identify and evaluate global trends in myopia research over the last century. They found that before 1990, this field of research did not receive much attention. Since 1991, however, the number of published articles gradually grew from 100 publications to more than 400 after 2011. In 2020, there were 822 articles published, and in 2021, there were 429 articles published until June, with the second most cited article from 2010. In it, Nicka et al. [44] performed a review of the anatomical structure of the choroid and its main functions, concluding that the thickening of the choroid may be mechanically associated with the scleral synthesis of macromolecules. Therefore, it may play an important role in the homeostatic control of ocular growth, and consequently in the aetiology of myopia and hyperopia. The year 2021 was considered a key year given its high number of publications and because it was the most cited. In that year, one publication that stands out is the one by Brennan et al. [45], in which the authors carried out a review of the literature on the effectiveness of myopia control methods, highlighting that despite the presence of many publications dedicated to the subject, little attention has been devoted to the understanding of “efficacy” in the control of myopia and its application. The effect of treatment has been expressed in multiple ways, making it difficult to compare therapies and prognoses for an individual patient. Available efficacy data are generally limited to two to three years, making the long-term effect of treatment uncertain.

As far as collaboration between countries is concerned, it has become one of the main patterns of scientific research between countries. In this study, it has been found that the United States is the main country that conducts research on complications associated with myopia. The connections between countries show that the United States attaches great importance to exchange and cooperation between scientific communities, which is related to the fact that it is the country with the most research production to date. On the other hand, it is also speculated that it has a greater advantage over other countries due to its better economy and spending on scientific research. However, it is equally important that numerous US authors have produced high-quality research with good communication and collaboration with other authors. For example, T.L. Young of the University of Wisconsin System carried out a study in which the genetic heterogeneity of myopia was confirmed. To do so, a locus for autosomal dominant pathological high myopia was mapped at 18p11.31. In addition, a significant association of high myopia with a second locus was reported in the 12q21–23 region. The identification of this gene may provide an insight into the pathophysiology of myopia and ocular development [46]. However, in terms of citations, K. Ohno-Matsui of the Graduate School of Medical and Dental Sciences in Tokyo was the most cited author and the one with the greatest collaboration with other authors. Thus, it has been found that there is cooperation between the three most cited authors. One of the studies with the most impact was the most cited publication in Group 2 [35]. The authors also demonstrated the efficacy and safety of ranibizumab 0.5 mg compared to photodynamic therapy with verteporfin in patients with visual impairment due to myopic choroidal neovascularisation [47].

Journals allow researchers to obtain information in order to select the most suitable ones in which to publish their articles. In this study, it has been found that the ten most active journals published 41.3% of the total number of publications on complications of myopia, which indicates that there is a distribution of the literature among various journals, possibly due to the numerous areas of research. Therefore, on the one hand, researchers may have many journal options, and on the other, they may have difficulty choosing the most suitable journal due to the lack of knowledge or experience [48]. However, a concordance rate of 70% was found between the ten most active journals and the ten most cited journals, which suggests the good quality of research in this field.

Regarding the main keywords, “myopia”, “prevalence” and “eyes” stand out. As shown in this study, this is related to the fact that the main research topic is the importance of axial growth in myopic subjects. This is related to the greater precision of axial length measurements together with the observation that future pathology in the high myopic eye is associated with excessive axial lengthening. For this reason, it has been concluded that all clinical trials for the control of myopia must have as an objective the reduction in axial growth [49]. In turn, visual impairment is more associated with axial length than with refractive error [50]. Most interventions show a reduction in both myopia progression and axial length [51]. In addition, this group is the one that presents the most interest, since the increase in axial length is strongly associated with a variety of diseases [12,42,43,44,45,46,47,48,49,50,51,52,53,54], including myopic maculopathy, open-angle glaucoma, posterior subcapsular cataract and retinal detachment [12]. Regarding axial growth in children, there is still no clear consensus. In the CLEERE study, it has been found that the greatest axial growth took place one year before myopia appeared. Thus, myopic futures grew 0.33 mm, decreasing to 0.20–0.27 per year after onset and during myopia progression. This was not a myopia control study, so it involved children with corrected simple vision [53]. Subsequently, in that same study, it was shown that a change in axial length of 0.22 mm per year was an indication of a greater progression of myopia. In turn, it was independent of the history of axial or refractive progression of the previous year [54].

Thus, it has been concluded that axial length increases in myopic children around 0.3 mm per year in younger children and 0.2 mm per year in older children.

Another subject of great interest is whether there are differences based on ethnicity. Thus, Tideman et al. [55] found that 9-year-old European myopes experienced 0.34 mm of axial elongation in one year, with a range of 0.17 to 0.53 mm.

Similarly, Rozema et al. [56] found that 7- to 9-year-old Asian ethnic progressive myopes experienced axial growth of at least 0.3 mm per year, declining to around 0.2 mm per year until the end of their term measurement from 12 to 13 years old.

In the group of myopic maculopathy, the publication by Ohno-Matsui et al. [35] was the most cited. In this study, the authors developed a classification system for myopic maculopathy. They defined five categories: “without myopic retinal degenerative lesion” (Category 0), “tessellated fundus” (Category 1), “diffuse chorioretinal atrophy” (Category 2), “patchy chorioretinal atrophy” (Category 3), and “macular atrophy” (Category 4). Recently, this classification was questioned by various authors. Thus, Ruiz Medrano et al. [57] reported that a more comprehensive staging system is needed as disease staging needs to be more precisely defined to improve follow-up by allowing physicians to more accurately monitor changes over time. This is very important due to the progression of this disease. In turn, by unifying the classification systems, it will allow the establishment of standardisation classification criteria as well as the comparison of the findings between various multicentric and international studies. Subsequently, Parolini et al. [58] created a new classification based on all stages of Myopic Traction Maculopathy that offers information on the pathogenesis and natural history of the disease.

In Group 3, the most cited publication was by Chen et al. [36]. In this publication, the authors reviewed the risk of developing cataracts after the implantation of phakic intraocular lenses in myopic patients. The incidence of cataract formation was 1.29%, 1.11%, and 9.60% with anterior chamber, iris-fixed, and posterior chamber IOLs, respectively. Furthermore, in regard to patients who already had cataracts, their progression increased by 29.5%. The results of this study are consistent with those of numerous studies. Thus, an association has been found between cataract surgery and retinal detachment in myopic patients [23].

In Group 4, Mitchell et al. [37] quantified the relationship between myopia and open-angle glaucoma, ocular hypertension, and intraocular pressure in the elderly population. The results showed that myopic patients are under a two to three times greater risk of developing glaucoma compared to non-myopic patients. This agrees with current evidence, so Ha et al. [59] showed that for each dioptre increase in myopia, the risk of glaucoma increases by about 20%. In turn, the risk increases faster in high myopia.

The importance of genetics in myopia, Group 5, is gaining more and more importance. Thus, Snead et al. [38] reviewed Sickler syndrome since it is generally associated with congenital but not progressive high myopia. Advances in molecular technologies (linkage analysis, candidate gene authentication, whole-genome association, and next-generation sequencing) revealed many myopia-related loci and genetic mutations or variants. Furthermore, there were also ethnic differences in the occurrence of myopia, and different genetic components were associated with the development of myopia-related phenotypes. Therefore, a better understanding of the genetic basis that triggers and controls myopic changes may further aid in myopia prevention.

In Group 6, Lim et al. [39] evaluated the association between myopia and the development of cataracts. The results demonstrated that early-onset myopia may be a risk factor in the development of posterior subcapsular cataracts. Nuclear cataract was associated with acquired myopia, and high myopia was associated with all three types of cataracts. Since cataract is one of the main causes of blindness in the world, studies currently focus on studying how to prevent it and the risks it causes [60,61].

In the last group, Quinn et al. [40] analysed the risk factors associated with retinopathy in preterm babies [62]. Retinopathy of prematurity is a leading cause of childhood blindness. The incidence of retinopathy of prematurity varies from country to country [63]. Several risk factors such as low birth weight, high oxygen saturation, low gestational age, intraventricular haemorrhage, respiratory distress syndrome, phototherapy, sepsis, mechanical ventilation, anaemia, patent ductus arteriosus, broncho pulmonary dysplasia, blood transfusion, necrotising enterocolitis, and low Apgar scores have been associated with the development of ROP [64,65,66,67].

Low birth weight and increased severity of retinopathy in preterms were strong predictors of myopia and high myopia. In addition, anisometropia, astigmatism and the presence of posterior pole debris were also associated with a higher incidence of myopia and high myopia. The most recent research has focused on the various forms of treatment and their complications. Thus, Marlow et al. [68] showed that after two years of treatment with ranibizumab 0.2 mg, a reduction in high myopia was shown, possibly with a better vision-related quality of life. This treatment did not appear to affect non-ocular infant development. On the other hand, Barnett et al. [69] showed that in the most severe cases, a poor outcome can be favoured with anti-VEGF. In less severe cases, the risk of recurrence and the need for additional treatments may favour laser.

These results offer an instructive perspective on the research that currently exists and the possible future direction in regard to complications in myopia. This article allows researchers to consider the current situation and the development trend of research in the field of complications associated with myopia, as well as the most active areas and those of the greatest interest to other researchers. This knowledge will help them quickly identify collaborators in the field of ocular complications associated with myopia from the perspective of spatial distribution. On the other hand, this study will also help researchers to identify important references and journals that are productive and influential. In other words, the results of this study can help researchers choose suitable collaborators or journals to promote their research as well as obtain information on the main research topics of most interest today. In addition, it allows a joint perspective of those areas in which there is more knowledge and, conversely, those other areas in which more research is still necessary to be able to reach precise conclusions.

## 5. Conclusions

This study has provided a comprehensive, objective analysis on the different research fields in complications in myopia.

It has been seen that it is an expanding area of research, with growth in recent years and the possibility of very broad new lines of research.

Through citation network analysis, we determined that the most cited publication analysed myopia prevalence worldwide, in which the high rates in East Asia stand out.

On the other hand, the seven main groups indicated what the main research topics were in this field. The most relevant one was the importance of axial growth in myopic patients.

## Figures and Tables

**Table 1:** Top 10 institutions with the highest number of publications.

Category	Frequency	Centrality	Degree	Half-Life
University of California System	325	0.00	79	6.5
National University of Singapore	288	0.00	110	12.5
Singapore National Eye Center	286	0.00	88	14.5
University of London	255	0.00	3	−0.5
Sun Yat Sen University	249	0.00	67	12.5
Tokyo Medical Dental University	218	0.00	59	17.5
University College London	209	0.00	6	−0.5
Ruprecht Karls University Heidelberg	196	0.00	5	2.5
Fudan University	191	0.00	31	8.5
Eberhard Karls University of Tubingen	182	0.00	4	−0.5

**Table 2:** Top 10 journals with the most publications.

Journal	Total Publications	Impact Factor (2021)	Quartile Score	SJR (2021)	Citations/Docs (2 Years)	Total Citations (2021)	H Index	Country
*Investigative Ophthalmology Visual Science*	882	4.925	Q1	1.399	4.054	8056	229	United States
*Journal of Cataract and Refractive Surgery*	482	3.528	Q2	1.367	1.722	2297	148	United States
*American Journal of Ophthalmology*	366	5.488	Q1	2.301	4.100	5077	194	United States
*Ophthalmology*	346	14.2777	Q1	4.412	5.209	7102	256	United States
*Retina The Journal of Retinal and Vitreous Diseases*	326	3.975	Q2	-	-	-	-	United States
*British Journal of Ophthalmology*	263	5.908	Q1	1.800	4.910	4882	162	United Kingdom
*Graefes Archive for Clinical and Experimental Ophthalmology*	249	3.535	Q2	1.305	3.504	3494	105	Germany
*Optometry and Vision Science*	239	2.106	Q3	0.561	1.747	871	102	United States
*Eye*	191	4.456	Q1	1.427	3.567	3749	106	United Kingdom
*Journal of Refractive Surgery*	162	3.255	Q1	1.298	2.758	1059	99	United States

**Table 3:** Top 20 most-used keywords.

Keyword	Frequency	Degree	Total Link Strength
myopia	1435	91	20,193
prevalence	1085	88	8660
eyes	934	87	6389
optical coherence tomography	611	62	26,272
children	536	96	4579
risk factors	524	76	4105
pathologic myopia	497	66	4080
refractive error	465	117	2705
axial length	459	107	5659
eye growth	456	129	4243
population	445	61	3334
glaucoma	426	78	5042
in situ keratomileusis	409	86	2639
open-angle glaucoma	387	95	2994
progression	392	74	3261
surgery	379	69	2641
high myopia	378	69	6553
form-deprivation myopia	364	91	3190
macular degeneration	360	68	2680
eye	356	87	2682

## Data Availability

Not applicable.
